# Epidemiology and Outcomes of Complicated Skin and Soft Tissue Infections among Inpatients in Southern China from 2008 to 2013

**DOI:** 10.1371/journal.pone.0149960

**Published:** 2016-02-26

**Authors:** Xiaoyan Li, Yunqin Chen, Weiguo Gao, Wenwei Ouyang, Jia Wei, Zehuai Wen

**Affiliations:** 1 Key Unit of Methodology in Clinical Research, Guangdong Provincial Hospital of Chinese Medicine, 111 Dade Road, Guangzhou 510120, China; 2 R & D information China, AstraZeneca, 199 Liangjing Road, Pudong, Shanghai, 201203, China; Rockefeller University, UNITED STATES

## Abstract

Complicated skin and soft tissue infections (cSSTI) are some of the most commonly treated infections in hospitals, and place heavy economic burdens on patients and society. Here we report the findings from an analysis of cSSTI based on a retrospective study which was conducted within the Chinese inpatient population. We focused our research on the analysis of the patient population, antibiotic treatment, clinical outcome and economic burden. The study population comprised 527 selected patients hospitalized between 2008 and 2013. Among the hospitalizations with microbiological diagnoses, 61.41% (n = 113) were diagnosed as infected with Gram-positive bacteria, while 46.20% (n = 85) were infected with Gram-negative bacteria. The most commonly found Gram-positive bacteria was *Staphylococcus aureus* (40.76%, n = 75), and the most common Gram-negative bacteria was *Escherichia coli* (14.13%, n = 26). About 20% of the *Staphylococcus aureus* were methicillin-resistant. The resistance rate of isolated *Staphylococcus aureus* or *Escherichia coli* to penicillin was around 90%; in contrast, the resistance rate to vancomycin, linezolid or imipenem was low (<20%). A large percentage of patients were treated with cephalosporins and fluoroquinolones, while vancomycin and imipenem were also included to treat drug-resistant pathogens. Over half of the hospitalizations (58.43%, n = 336) experienced treatment modifications. The cost to patients with antibiotic modifications was relatively higher than to those without. In conclusion, our study offers an analysis of the disease characteristics, microbiological diagnoses, treatment patterns and clinical outcomes of cSSTI in four hospitals in Guangdong Province, and sheds lights on the current clinical management of cSSTI in China.

## Introduction

Complicated skin and soft tissue infections (cSSTI) are associated with remarkable morbidity, as they make up some of the most commonly treated-infections in hospitals [1–3]. Although previous reports have discussed the risk factors for cSSTI somehow a concensus has not been reached on this issue [4, 5].

cSSTI has many classifications based on the infection type, its microbial etiology or severity. The majority of the pathogens leading to cSSTI are aerobic Gram-positive cocci, especially *Staphylococcus aureus* [6, 7], however, cSSTI could be attributed to multiple bacteria, such as aerobic and anaerobic pathogens [8, 9]. Among the pathogens accounting for cSSTI, methicillin-resistant *Staphylococcus aureus* (MRSA) has garnered special attention, not only because of its high prevalence, but because infection by this bacterium also leads to poor clinical outcomes [10].

Currently there is no consensus on how to manage these infections, but cSSTI often requires intravenous antibiotic therapy, surgical intervention, or both [1]. Therefore, the selection of antibiotics plays an essential role in the management of cSSTI [11]. However, it often lacks a confirmed microbiological diagnosis when treatments begin, making such selections complicated. It is noted that inappropriate initial antibiotic treatments may result in unfavorable clinical outcomes, consequently identifying risk factors for inappropriate initial treatments becomes an important task in the clinical management of cSSTI. Since the resistance to antibiotics has greatly increased in clinical practice, treatment of these infections has become more challenging [12, 13]-the war against MRSA offers the best example of this phenomenon. In addition, multidrug-resistant and extensively drug-resistant Gram-negative pathogens, such as *Klebsiella pneumoniae*, have also posed threats in many countries [14].

A further analysis of the disease characteristics, microbiological diagnoses, treatment patterns and clinical outcomes of cSSTI is needed. One previous European study on cSSTI, The Retrospective study to Assess the Clinical Management of Patients with Moderate-to-Severe cSSTI in the Hospital Setting (REACH: NCT01293435), has provided relevant information for understanding of above-mentioned fields of cSSTI [15]. However, only a few reports on cSSTI have been issued in Asia. Here we report the findings from an analysis of cSSTI based on a retrospective study which was conducted among the Chinese in-patient population. The primary objective of this study is to collect and analyze data from the electronic medical records of Chinese cSSTI patients, and provide insights into the clinical diagnosis and management of cSSTI, all of which is critical to improve clinical outcomes and reduce the economic burden posed to society.

## Materials and Methods

This study is a retrospective, non-interventional study designed to assess the disease characteristics, microbiological diagnoses, treatment patterns and clinical outcomes of adult patients hospitalized with cSSTI.

All the data were collected from electronic medical records (EMRs) acquired from four hospitals (Dade, Fangcun, University City and Ersha Island) of a healthcare conglomerate, the Guangdong Provincial Hospital of Chinese Medicine. Established in 1933 and headquartered in the downtown area of Guangzhou city, Guangdong Province in southern China, the organization is the largest healthcare conglomerate practicing both Chinese and Western medicine in China with 3140 beds, and has over 5 million outpatients and 70,000 inpatients each year. Being a leading healthcare organization in clinical research in China, the underlying hospitals established EMR systems in 2003 and became the earliest EMR adopters among all the hospitals in China. The study was approved by the ethical committee of the Guangdong Provincial Hospital of Chinese Medicine. Due to the retrospective nature of the study, informed consent was not deemed necessary. All the patient data were anonymized and de-identified prior to analysis.

### Patients

The study population comprised patients diagnosed with cSSTI hospitalized between 2008 and 2013 in four hospitals (Dade, Fangcun, University City and Ersha Island); they were diagnosed by physicians according to criteria set forth in the Practice Guidelines for the Diagnosis and Management of Skin and Soft-Tissue Infections [16], including infections with local/systemic presentations. Finally 527 patients with primary diagnosis of cSSTI in 575 hospitalizations were eligible for inclusion, and the EMR data from this population were reviewed and extracted for analysis.

### Pathological diagnosis

Microbiological tests were applied to indentify bacteria according to CLSI procedure (Clinical and Laboratory Standards Institute. Abbreviated identification of bacteria and yeast; approved guideline. In: Document M35-A2, 2^nd^ ed.). These tests are based on Gram’s staining, observing bacterial morphotypes under a microscope, cultural characteristics in the culture medium, and various specific biochemical reactions. Bacterial species can be identified according to their unique activities, such as physiological reactions and metabolites. The main method used in these hospitals was an automatic bacterial identification and susceptibility analysis system (Microscan Walkaway 96 Plus). Antibiotic resistance testing was also performed according to CLSI procedure. Penicillins (Ampicillin, Piperacillin, Piperacillin/tazobactam), third-generation cephalosporins (Ceftriaxone, Cefoperazone, Cefoperazone/sulbactam, Ceftazidime, Ceftazidime), the fourth-generation cephalosporin Cefepime, the carbapenem Imipenem, the monobactam Aztreonam, the fluoroquinolone (Ciprofloxacin, Levofloxacin), and aminoglycosides (Amikacin, Gentamicin) were used for resistance testing. Methods for dilution antimicrobial susceptibility tests for bacteria that grow aerobically: approved standard. In: Document M07-A9, 9^th^ ed.), and microorganisms were tested on a susceptibility panel. In addition, the empirical antibiotic treatment principle is based on Antibacterial Drug Selection Guidelines established by the Chinese Medical Association.

### Study variables

Demographic information including the date of birth, date of hospitalization and discharge, sex, education level, occupation, medical insurance, and marital status were extracted from the EMRs. History of previous medical conditions, events, and life styles were extracted from corresponding parts of the EMRs. The study measures assessed included patient characteristics, pathogen characteristics, antibiotic susceptibility, laboratory tests and clinical outcomes (length of stay [LOS], readmission and mortality). All analyses were descriptive in nature.

### Statistical analysis

All the descriptive analysis was conducted using R3.1.1 (http://www.cran.r-project.org/web/packages/). The mean differences of continuous variables between two groups were tested with the Student’s t-test or the Wilcoxon rank sum test when appropriate. A Fisher’s Exact Test was employed to test the differences in categorical variables between groups. P < 0.05 was considered statistically significant.

## Results

### Patient characteristics and microbiological diagnoses

527 patients (575 hospitalizations, due to recurrence cases) were admitted to four hospitals (Dade, Fangcun, University City and Ersha Island) between 2008 and 2013, with a mean age of 56.14 years. 9.13% (n = 225) patients were ≥65. The degree of co-morbidity with hypertension is relatively high (27.65%, n = 159), followed by diabetes (17.74%, n = 102) and cancer (17.04%, n = 98) (Table 1). The data about medication during treatment is also shown in Table 1. As to medical history, a considerable proportion of hospitalizations received surgical treatment (30.43%, n = 175) or received medication (25.57%, n = 147) prior to hospitalization, among which 29 inpatients (5.04%) were treated with anti-diabetes medications (Table 1). During hospitalization, 175 (36.17%) received surgical treatment. Among the 575 hospitalizations, 92 (16%) were diagnosed with healthcare-associated infection (HAI).

**Table 1:** Baseline patient demographics, characteristics and medical history.

Hospitalizations with cSSTI	N = 575
Number of patients (n)	527
Age, years, mean ±SD [median]	56.14 ± 20.11 [58]
> = 65 years, n (%)	225 (39.13)
Gender, n (%)	
Male	308 (53.57)
Surgical treatment during hospitalization, n (%)	208 (36.17)
Medication during treatment, n (%)	
Antibacterial antibiotics	553 (96.17)
Cardiac therapy	301 (52.35)
Anticoagulants	258 (44.87)
Antipruritics, including antihistamines, anesthetics, etc.	241 (41.91)
Anti-inflammatory and antirheumatic products	218 (37.91)
Drugs used in diabetes	138 (24.00)
Antineoplastic agents	123 (21.39)
Immunostimulants / Immunosuppressants	76 (13.22)
Surgical treatment prior to hospitalization, n (%)	175 (30.43)
Hospitalized patients with a microbiological diagnosis, n (%)	184 (32.00)
Patients diagnosed with HAI, n (%)	92 (16.00)
Medication prior to hospitalization, n (%)	147 (25.57)
Antibacterials for systemic use	11 (1.91)
Corticosteroids, dermatological preparations	5 (0.87)
Drugs used in diabetes	29 (5.04)
Antineoplastic agents	4 (0.70)
Cardiac therapy	9 (1.57)
Co-morbidities, n (%)	
Hypertension	159 (27.65)
Diabetes	102 (17.74)
Cancer/Malignancy	98 (17.04)
Fatty liver	75 (13.04)
Hyperglycemia	59 (10.26)
Renal failure	35 (6.09)
Heart failure	21 (3.65)
Liver failure	3 (0.52)
Type of lesion^a^	
Cellulitis/fasciitis/erysipelas	102 (17.57)
Abscess	46(8.01)
Post-surgical wound	91 (15.83)
Decubitus ulcer	36 (6.26)
Peripheral vascular disease ulcer	11 (1.91)
Post-traumatic wound	8 (1.39)
Eczema	12 (2.09)
Burn/bite	6(1.04)
Necrosis	4 (0.70)
Unknown	280 (48.70)

HAI, healthcare-associated infection

^a^ One hospitalization could have more than one type of cSSTI lesions.

Microbiological test results (see Table 2) were only available for 184 (32.5%) hospitalizations in total. Of the hospitalizations with a microbiological diagnosis, 61.41% (n = 113) were diagnosed as infected with Gram-positive bacteria, while 46.20% (n = 85) were infected with Gram-negative bacteria. The most commonly found Gram-positive bacteria was *Staphylococcus aureus* (40.76%, n = 75), and 20% of the *Staphylococcus aureus* were methicillin-resistant. Among the Gram-negative bacteria *Escherichia coli* was most prevalent (14.13%, n = 26). For the patients infected with Enterobacteriaceae, the incidence of *Klebsiella pneumoniae* (6.52%, n = 12) was also relatively high. There were 33 hospitalizations given a “mixed infection” diagnosis and 22 were infected with two bacteria, among which 4 were confirmed to have *Staphylococcus aureus* & *Escherichia coli* infections, and 3 had *Pseudomonas pyocyaneum* & *Staphylococcus aureus* infections (data not shown).

**Table 2:** Microbiological diagnoses.

Pathogen(s)	Hospitalizations, n (%) (N = 184)
Gram-positive bacteria	113(61.41)
Staphylococcaceae spp.	
*Staphylococcus aureus*	75(40.76)
MRSA	14(7.61)
MSSA	61(31.15)
VRSA	0(0.00)
VSSA	75(40.76)
*Staphylococcus haemolyticus*	7(3.80)
*Staphylococcus epidermidis*	3(1.63)
MRSE	2(1.09)
MSSE	1(0.54)
Enterococcaceae spp.	
*Enterococcus faecalis* (Group D)	9(4.89)
*Enterococcus faecium* (Group D)	5(2.72)
Gram-negative bacteria	85(46.20)
Enterobacteriaceae spp.	
*Escherichia coli*	26(14.13)
*Klebsiella pneumoniae*	12(6.52)
*Enterobacter cloacae*	9(4.89)
*Proteus mirabilis*	9(4.89)
*Klebsiella oxytoca*	3(1.63)
*Morganella morganii*	3(1.63)
Moraxellaceae spp.	
*Acinetobacter baumannii*	6(3.26)
Pseudomonadaceae spp.	
*Pesudomonas pyocyaneum*	5(2.72)
*Pseudomonas aeruginosa*	15(8.15)
Xanthomonadales spp.	
*Stenotrophomonas maltophilia*	3(1.63)

MRSA, methicillin-resistant *Staphylococcus aureus;* MSSA, methicillin-sensitive *Staphylococcus aureus;* VRSA, vancomycin-resistant *Staphylococcus aureus*; VSSA, vancomycin-sensitive *Staphylococcus aureus*; MRSE, methicillin-resistant *Staphylococcus epidermidis*; MSSE, methicillin-sensitive *Staphylococcus epidermidis*.

### Antibiotic resistance and medication treatment

The antibiotic resistance patterns of *Staphylococcus aureus*, *Escherichia coli*, *Klebsiella pneumoniae*, *Enterobacter cloacae* and *Pseudomonas aeruginosa* were also calculated. The resistance rate of isolated *Staphylococcus aureus* to penicillin was 89.8% (n = 44) and 93.02% (n = 80) during 2008–2010 and 2011–2013, respectively; resistance to erythromycin was 48.98% (n = 24) and 36.05% (n = 31), respectively. For antibiotics other than these two, the resistance rate was well below 20%. All patients treated with vancomycin and imipenem were sensitive to the treatment (see Table A in S1 File and Fig B in S1 File).

The resistance rate of isolated *Escherichia coli* to ampicillin was 96.15% (n = 25) and 100% (n = 20) in the periods of 2009–2010 and 2011–2013 respectively; the rate was also above 80% between 2009 and 2010 for piperacillin, aztreonam, ceftriaxone, ceftazidime, and cefepime. All patients treated with imipenem and amikacin were sensitive to the treatment between 2011 and 2013 (Table B in S1 File and Fig B in S1 File). The resistance rate of isolated *Klebsiella pneumoniae* to ampicillin was 100% (n = 15) during 2010–2013. In contrast to the high resistance rate (>50%) for ampicillin, sulbactam, aztrenam, ceftazidime, and gentamicin, all patients (infected with *Klebsiella pneumoniae*) treated with imipenem between 2010 and 2013 were sensitive to this drug (Table C in S1 File and Fig B in S1 File). The resistance rate of isolated *Enterobacter cloacae* to ampicillin was also 100% (n = 15) between 2010 and 2013. It was still apparent that all patients (infected with *Enterobacter cloacae*) treated with imipenem were sensitive to this antibiotic, and also true for amikacin (Table D in S1 File and Fig B in S1 File). The data regarding the resistance rate of patients infected with *Pseudomonas aeruginosa* are also detailed in Table E in S1 File and Fig B in S1 File.

Antibiotic treatment information, including initial and overall treatment patterns, are detailed in Table 3. A large percentage of patients were treated with cephalosporins, including 1st generation, 2nd generation and 3rd generation drugs. The most frequently used cephalosporins for mono-threapy were cefoperazone and sulbactam (9.57%,) and cefuroxime (8.35%), accounting for their overall treatment. Moreover, 57 hospitalizations (9.91%) received levofloxacin. Cefoperazone and Tazobactam combined with Ornidazol was used as a top combination therapy in both initial and overall patterns while imipenem combined vancomycin was only used in overall treatment. Regardless of treatment pattern, the general antibiotics usage was list in Table F in S1 File. In all, levofloxacin, and Cefoperazone and Sulbactam were the most common used medication for cSSTI. Besides, imipenem,vancomycin or norvancomycin, meropenem were also used for multiple drug-resistant patients (Table F in S1 File).

**Table 3:** Most frequently used antibiotics therapy pattern for initial treatment and overall treatment.

Antibiotic therapy pattern	Initial treatment,n (%)	Overall treatment,n (%)
Mono-therapy		
**Cephalosporin 3rd generation**	126 (21.91)	221 (38.43)
Cefoperazone and Sulbactam	35 (6.09)	55 (9.57)
Ceftriaxone and Tazobactam	27 (4.70)	33 (5.74)
Cefoperazone and Tazobactam	26 (4.52)	42 (7.30)
Ceftizoxime	17 (2.96)	27 (4.70)
Cefodizime	10 (1.74)	20 (3.48)
Ceftriaxone	9 (1.57)	21 (3.65)
Cefixime	2 (0.35)	23 (4.00)
**Cephalosporin 2nd generation**	83 (14.43)	107 (18.61)
Cefamandole	33 (5.74)	40 (6.96)
Cefuroxime	33 (5.74)	48 (8.35)
Cefotiam	11 (1.91)	12 (2.09)
Latamoxef	6 (1.04)	7 (1.22)
**Fluoroquinolones**	30 (5.21)	76 (13.22)
Levofloxacin	25 (4.35)	57 (9.91)
Ciprofloxacin	5 (0.87)	19 (3.30)
**Cephalosporin 1st generation**	44 (7.65)	60 (10.43)
Cefathiamidine	25 (4.35)	32 (5.57)
Cefazolin	19 (3.30)	28 (4.87)
**Penicillin combination**	8 (1.39)	39 (6.79)
Nitrofurazone	6 (1.04)	17 (2.96)
Piperacillin and Sulbactam	2 (0.35)	22 (3.83)
**Aminoglycoside**	12 (2.09)	34 (5.91)
Gentamicin	8 (1.39)	21 (3.65)
Amikacin	4 (0.70)	13 (2.26)
**Carbapenems**	4 (0.70)	19 (3.30)
Imipenem Cilastatin	4 (0.70)	19 (3.30)
**Lincomycin**	9 (1.57)	15 (2.61)
Clindamycin	9 (1.57)	15 (2.61)
**Combinationa therapy**		
Cefoperazone and Tazobactam+Ornidazole	5 (0.87)	12 (2.09)
Gentamicin+Levofloxacin	2(0.35)	10(1.74)
Imipenem Cilastatin+Vancomycin	0 (0)	10(1.74)
Imipenem Cilastatin+Nitrofurazone	2(0.35)	9 (1.57)

Mono-therapy: one antibiotics one time; Combination therapy: more than one antibiotics used one time.

In addition, over half of the hospitalizations (58.43%, n = 336) experienced treatment modifications.

### Clinical outcome

Clinical outcomes were detailed in Table 4. On average, the length of stay (LOS) was 18.9 days. The information about the distribution of hospitalizations according to length of stay is shown in Fig A in S1 File. In addition, 12 hospitalizations (2.08%) were admitted to the Intensive Care Unit (ICU) for management. Among all the hospitalizations, the majority (97.57%, n = 561) were eventually discharged from the hospitals. The mortality rate was 2.43% (n = 14), however, none of deaths were attributed to cSSTI. According to the follow-up data, there were 35 (6.64%) patients with recurrences of cSSTI. Among them, 20 (3.80%) patients with recurrence cases happened within three months after discharge.

**Table 4:** Clinical outcomes.

Clinical outcomes	N = 575
Treatment modifications, n (%)	336(58.43)
Length of stay, days, mean ±SD [median]	18.88 ± 24.99 [13]
Discharged from hospital, n (%)	561 (97.57)
Mortality rate, n (%)	12(2.43)
Mortality rate caused by cSSTI, n (%)	0 (0.00)
Reinfection/Recurrence, n (%)	35 patients (6.64)
< = 3 months	20 patients (3.80)
ICU admission rate, n (%)	12(2.08)

ICU, intensive care unit.

It was not surprising that the patients infected with MRSA had a higher antibiotic modification rate (78.57%, n = 11), than those with MSSA (67.21%, n = 41). This difference was also demonstrated by LOS—37.93 days for MRSA and 18.89 days for MSSA (Table 5). Interestingly, patients with MRSA were significantly elder than those with MSSA (p = 0.03).

**Table 5:** Clinical outcomes according to MRSA in comparison with MSSA.

Disease characteristics	MRSA (n = 14)	MSSA (n = 61)	P value
Antibiotics modification^a^, n (%)	11(78.57)	41(67.21)	0.53
Length of stay^b^, days, mean ±SD [median]	37.93 ± 47.20 [19]	18.89 ± 13.57 [15]	0.12
Mortality rate^a^, n (%)	0 (0)	0 (0)	1
Cost^b^, US$, mean±SD [median]	5215.21 ± 8667.48 [1943.63]	2612.64 ± 2965.28 [2145.61]	0.39
Age^b^, years, mean ±SD [median]	62.50 ± 20.35 [59]	51.92 ± 17.63 [56]	0.03

^a^ Variables were tested using Fisher’s Exact Test while

^b^ variables were tested using one tailed,Wilcoxon rank sum test.

### Economic burden

The information about the economic burden of cSSTI is shown in Table 6. The total cost per hospitalized patient was 2727.57±4045.58 US$, and western medicines (1041±1989.03 US$) including antibiotics accounted for the largest proportion (38.2%) of the cost. In addition to western medicines, herbs cost 258.98±306.46 US$ on average; the remaining cost were constituted by examinations, treatment procedures, bed, and diagnostic testing fees. It is notable that the total cost varied between patients with antibiotic drug modifications (3736.44±5194.59 US$) and those without antibiotic drug modifications (1601.20±1467.12 US$). For patients with antibiotic drug modifications, the cost was almost two-fold higher. A similar distinction was also observed in patients infected with MRSA (5215.21±8667.48 US$) and those infected with MSSA (2612.64±2965.28 US$).

**Table 6:** Economic burden of hospitalizations with cSSTI.

Category, US$,mean|median [range]	All Hospitalizations	With antibiotic modification Treatment or not			MRSA or not		
		YES (N = 336)	NO (N = 217)	P value	YES (N = 14)	NO (N = 61)	P value
**Total**	2727.57 |1642.57[2.82–44567.79]	3736.44 |2053.98[40.31–44567.76]	1601.20|1272.86[2.82–13879.21]	<0.001	5215.21|1943.63[2.82–33205.76]	2612.64|2145.61[44.73–19752.49]	0.785
**Western medicine**	1041.30|524.57 [0–21412.2]	1508.98|678.05[3.15–21412.24]	524.69|388.30[0–3291.67]	<0.001	2629.23|704.94[0–21412.2]	1004.45|658.40[7.62–10403.6]	0.515
**Herbs**	258.98|49.18[0–2399.62]	303.27 |186.01[0–2399.62]	197.08|102.29[0–1093.17]	<0.001	220.45|102.65[2.82–952.55]	280.92|159.23[0–1306.36]	0.359
**Examination**	473.18|332.10[0–8135.65]	590.73|401.40[5.09–8135.65]	344.84|281.06[0–2619.76]	<0.001	525.61|395.69[0–1435.19]	465.34|387.29[7.09–2783.24]	0.590
**Treatment procedure**	756.41|304.50[0–17100.55]	1078.27|427.40[5.57–17100.55]	404.30|188.48[0–7648.55]	<0.001	1627.92|392.55[0–11156.2]	659.11|354.35[15.14–4283.48]	0.669
**Bed**	189.27|124.20[0–2954.84]	240.89|155.65[2.69–2954.84]	124.27|99.68[0–1122.58]	<0.001	200.25|157.26[0–700]	194.06|150.97[2.98–951.61]	0.883
**Diagnosis**	8.43|6.29[0–49.84]	10.29|7.98[0–49.84]	6.04|5.32[0–28.06]	<0.001	11.74 |9.19[0–30]	8.75|7.26[0.48–26.61]	0.287

The cost of different groups: patients with versus without antibiotic drug modifications; patients with MRSA versus MSSA were compared. Wilcoxon rank sum test is used for P value calculation.

## Discussion

Although there have been a few studies on cSSTI conducted in Asia, especially on antibiotic treatment for cSSTI [17–19], information about microbiological diagnoses and treatment patterns of these infections are quite limited in this region. As a result, it is difficult to perform a comparison of our data with other Asian studies. However, our retrospective study provides a comprehensive and unique analysis of the management of cSSTI in China, which can be compared with previous corresponding studies conducted in Europe and North America.

Consistent with findings in the earlier REACH study [15], our patient characteristics data demonstrated the patients were composed of older people with a median age of 56.14 years. It is not strange that the majority of them had age-related co-morbidities, however, the occurrence of hypertension was even higher than diabetes, reflecting general public health trends in China. The rate of surgical treatment prior to hospitalization was also obviously higher than the rate (14%) offered in REACH study [15]; in contrast, the rate of antibacterial treatment for systemic use prior to hospitalization was surprisingly low (1.91%, n = 11), which may indicate that the infection rate prior to this study was lower than in previous studies. More patients of our study (16%) were diagnosed with HAI than in the REACH study (10%) [15], indicating the heightened severity of infections by drug-resistant and mixed microorganisms in our study.

A minority of our hospitalizations had microbiological diagnoses (32%, n = 184), and these diagnoses were made based on the samples removed from various sources (skin, wound swabs, secretions, drain, pus, puncture fluid, catheter, tissue, synovial fluid and venous blood).

According to microbiological diagnosis in our study, the most common organism identified in cSSTI infection was *Escherichia coli*, different from European studies where *Staphylococcus aureus* was often the most commonly found bacterium (27.9% for methicillin-sensitive *Staphylococcus aureus* and 10.2% for methicillin-resistant *Staphylococcus aureus* in REACH study) [4, 5, 15]. This probably indicates that in Chinese hospitals, infection with *Staphylococcus aureus*, especially methicillin-resistant *Staphylococcus aureus*, is not as common as in European hospitals. Moreover, it is not strange that our patient population was also affected by *Klebsiella pneumoniae*, and *Enterobacter cloacae*, which have drawn broad attention as pathogens for nosocomial infection in Europe and U.S. in recent years [20].

The successful management of cSSTI is involved in the fast identification of infectious agents and the appropriate use of antibiotic therapies, since the initial selection of antibiotics has an impact on the clinical outcomes of patients as well as costs. Generally, the initial selection of antibiotic treatments depends on the type and severity of the infection, and its suspected pathogens [21]. Given that, in this study, only a minority of patients had microbiological diagnoses, and there was no doubt that most patients did not have definite diagnoses of pathogens at the very beginning of hospitalization, almost all patients were treated empirically. As a result, initial antibiotic treatment modifications happened with over half of the patients, which could be considered high when compared with other studies (there was a rate of 39.6% for initial antibiotic treatment modifications in the REACH study) [5, 15]. Since reducing the risk among cSSTI patients would be beneficial for the successful management of these infections, it would be very helpful to conduct more studies to understand the risk factors associated with inappropriate initial antibiotic therapies in the future.

Resistance to antibiotics is another notorious issue in the management of cSSTI. For *Staphylococcus aureus* infections, penicillins (including penicillin, amoxicillin, oxacillin, etc.) are widely used for treatment in these hospitals, similar to findings in the REACH Study (18.3% of total patients were most often treated with amoxicillin-clavulanate initially; ampicillin and pipiracillin were also widely used) [15]. Because antibiotics have been prescribed for many years in clinics, it is understandable that the resistance rate to penicillin reflected in this report was specifically high. Since MRSA infection accounts for a proportion of *Staphylococcus aureus* infections, vancomycin and linezolid were also selected in our study, suggesting that clinicians have known that these two antibiotics should be included in MRSA treatments for some time. Nowadays a considerable proportion of cSSTIs are made up of MRSA infections, therefore, the empirical application of vancomycin and linezolid is suggested [22]. For *Escherichia coli* infection, penicillins and cephalosporins are widely used for treatment in these hospitals. Therefore, the resistance rate to cephalosporins was also high in our study, and the selection of penicillins or cephalosporins for treatment at the start of infection should be carefully examined in the future. Another issue we should specifically mention is the application of carbapenems for the treatment of cSSTI. Carbapenem resistance enterobacteriaceae infection has become commonplace in medical practice in recent years, and is a troublesome topic for clinicians [20]. However, in our study, none of isolated *Klebsiella pneumoniae* and *Enterobacter cloacae* were resistant to imipenem, which demonstrates that carbapenems in Chinese hospitals are still effective antibiotics against enterobacteriaceae.

Given that the length of stay (LOS) for patients is closely associated with hospital costs, it is often considered a key parameter of efficacy [23]. In our retrospective study, LOS was approximately 19 days, very similar to the number days (18.5 days) offered in the REACH study [15]. This indicates that the factors influencing LOS could be comprehensively similar between the two studies. However, in comparison with other previous reports, LOS was longer than expected [4, 5], suggesting that there were more severe infections and co-morbidities in our study. The mortality rate caused by cSSTI was 0%, and the reinfection and recurrence rate (6.96%) was also slightly lower (8.6%) than in the REACH study [15]—this demonstrated the relatively successful management of infection in this study’s patient cohort.

According to previous studies, the cost of treating cSSTI could be high if infections were associated with the presence of co-morbidities, the choice of specific antibiotic agents, and the requirement for initial antibiotic therapy modifications [24]. The increase in cost could be attributed to the switch in antibiotic regimens or the presence of complications or co-morbidities. Our study on the economic burden of treatment was focused on initial antibiotic treatment modifications, the need for which often suggests inadequate initial therapy. Compared with patients without antibiotic modifications, the cost for patients with these modifications was relatively high. As a result, reducing the economic burden was largely dependent on adequate initial antibiotic therapies, which require timely and correct microbiological diagnosis at the onset of cSSTI. On the other hand, the costs for MRSA patients was almost two fold higher than for MSSA patients on average, and western medicines (especially antibiotics) mainly contributed to the difference—this result was also in accord with findings in the previous study [24].

Our study has several limitations. First, the patients were selected from hospitals in one region; therefore the results may not be generalized to non-hospitalized patients, or applicable to hospitals in other regions in China. Second, the sample size is not as large as those in previous studies (such as the REACH study [15]), and the number of patients with microbiological diagnoses is especially low. As a result, the statistical power of our analysis may be limited. Third, although we rigorously reviewed the data, data accuracy and completeness largely depend on the judgment of clinicians in the participating hospitals.

In conclusion, our study provides analysis of the disease characteristics, microbiological diagnoses, treatment patterns and clinical outcomes of cSSTI in southern China. The data highlight the importance of accurate and timely microbiological diagnosis, appropriate initial antibiotic treatment, and the reasonable management of any economic burden. We believe that the Western management guidelines for cSSTI, such as the Practice Guides for the Diagnosis and Management of Skin and Soft-Tissue Infections by the Infectious Disease Society of America [16], should be modified if they are to be applied to China and other Asian countries, based on the distinct occurrence and resistance patterns of local pathogens. We also expect that our results, together with further research data, will be able to answer the questions related to the successful clinical management of cSSTI in the future.

## Supporting Information

S1 File Supplementary information including distribution of length of stay in hospitalization, antibiotic resistance patterns for six bacterium and cSSTI infection types and microbiology. (DOC)
