# Supplementary material for: Epidemiology and Outcomes of Complicated Skin and Soft Tissue Infections among Inpatients in Southern China from 2008 to 2013
Source: PLoS One. 2016 Feb 26;11(2):e0149960. doi: 10.1371/journal.pone.0149960 (PMC4769280; doi:10.1371/journal.pone.0149960)
Supplement: S1 File — (DOC) [file pone.0149960.s001.doc]

**Supplementary data**

**Figure A.Distribution of hospitalizations according to length of stay**


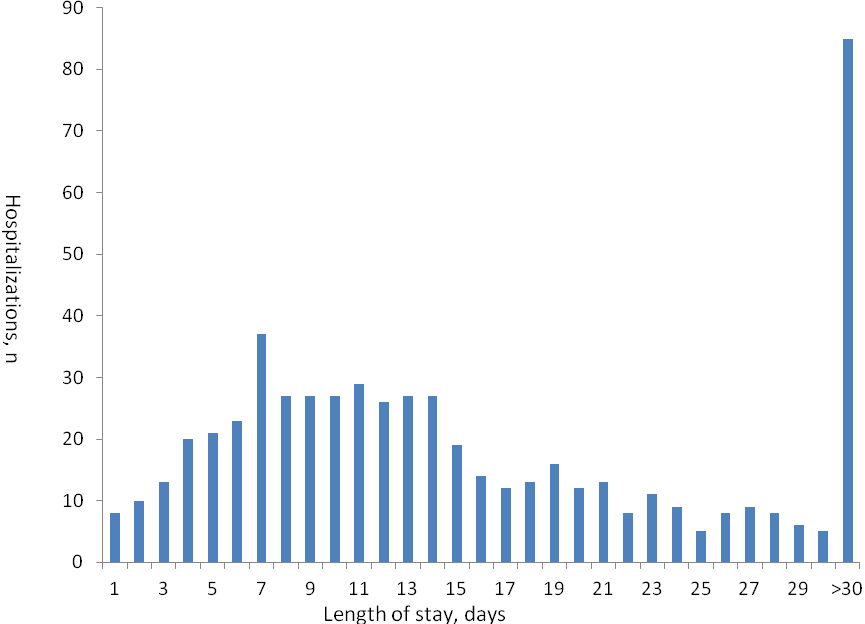


**Figure B. Antibiotic resistance patterns for *Staphylococcus aureus, Escherichia coli,* *Klebsiella pneumonia, Enterobacter cloacae* and *Pseudomonas aeruginosa***


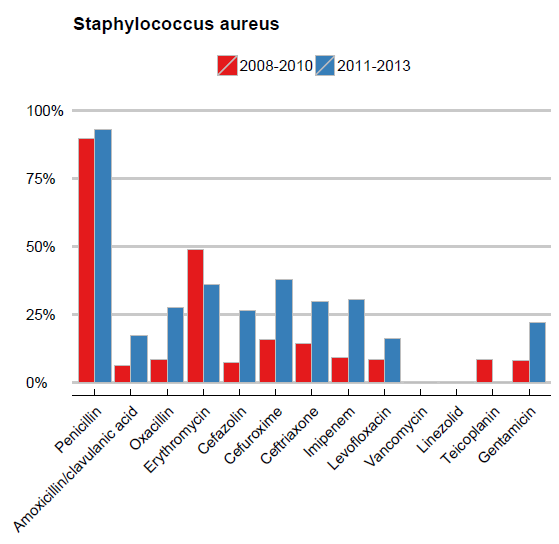

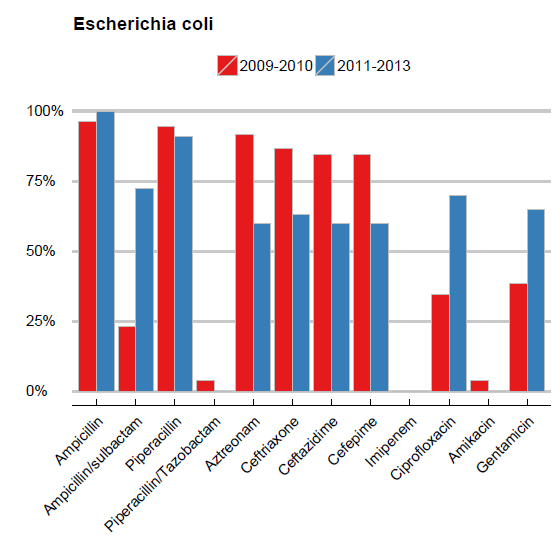


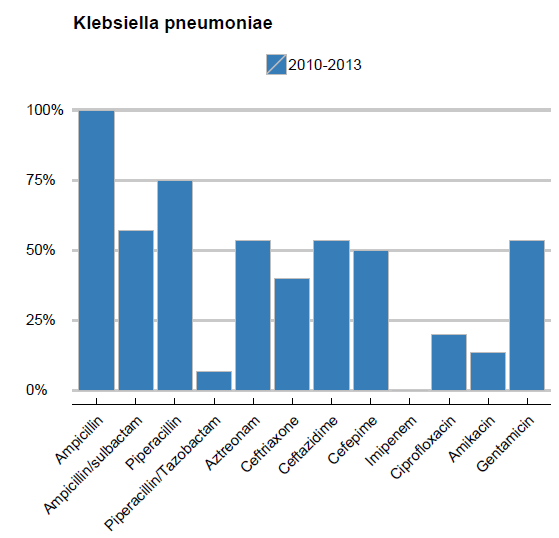

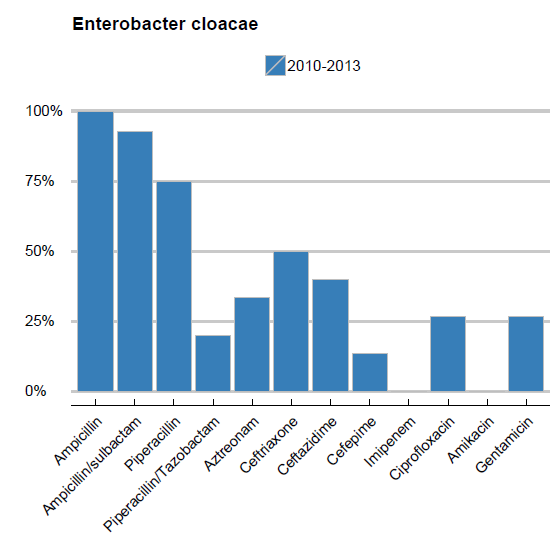


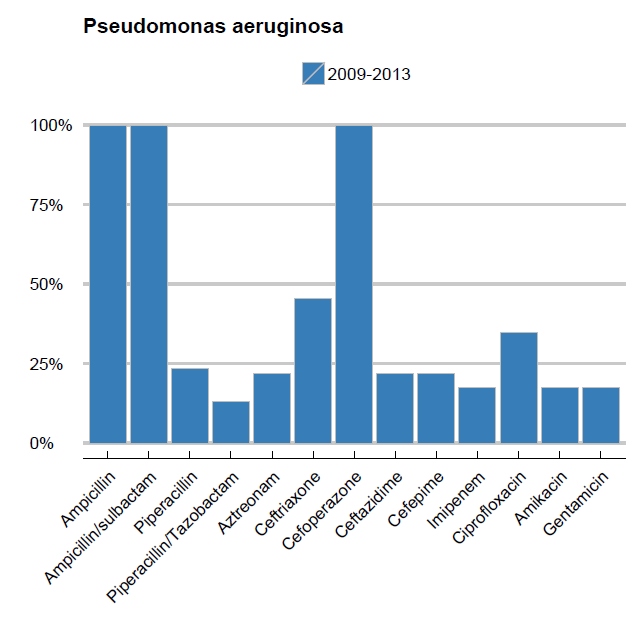


**Table A. Antibiotic resistance proportion of *Staphylococcus aureus***

| **Antibiotics** | **2008-2010** | | | **2011-2013** | | |
| --- | --- | --- | --- | --- | --- | --- |
|  | **total** | **resistance** | **Proportion**  **(%)** | **total** | **resistance** | **Proportion**  **(%)** |
| Penicillin | 49 | 44 | 89.8 | 86 | 80 | 93.02 |
| Amoxicillin/clavulanic acid | 32 | 2 | 6.25 | 70 | 12 | 17.14 |
| Oxacillin | 36 | 3 | 8.33 | 80 | 22 | 27.5 |
| Erythromycin | 49 | 24 | 48.98 | 86 | 31 | 36.05 |
| Cefazolin | 27 | 2 | 7.41 | 49 | 13 | 26.53 |
| Cefuroxime | 19 | 3 | 15.79 | 29 | 11 | 37.93 |
| Ceftriaxone | 21 | 3 | 14.29 | 37 | 11 | 29.73 |
| Imipenem | 33 | 3 | 9.09 | 72 | 22 | 30.56 |
| Levofloxacin | 24 | 2 | 8.33 | 80 | 13 | 16.25 |
| Vancomycin | 49 | 0 | 0 | 86 | 0 | 0 |
| Linezolid | 49 | 0 | 0 | 86 | 0 | 0 |
| Teicoplanin | 24 | 2 | 8.33 | 9 | 0 | 0 |
| Gentamicin | 49 | 4 | 8.16 | 86 | 19 | 22.09 |

**Table B. Antibiotic resistance proportion of *Escherichia coli***

| **Antibiotics** | **2009-2010** | | | **2011-2013** | | |
| --- | --- | --- | --- | --- | --- | --- |
|  | **total** | **resistance** | **Proportion**  **(%)** | **total** | **resistance** | **Proportion**  **(%)** |
| Ampicillin | 26 | 25 | 96.15 | 20 | 20 | 100 |
| Ampicillin/sulbactam | 26 | 6 | 23.08 | 18 | 13 | 72.22 |
| Piperacillin | 18 | 17 | 94.44 | 11 | 10 | 90.91 |
| Piperacillin/Tazobactam | 26 | 1 | 3.85 | 20 | 0 | 0 |
| Aztreonam | 24 | 22 | 91.67 | 20 | 12 | 60 |
| Ceftriaxone | 15 | 13 | 86.67 | 19 | 12 | 63.16 |
| Ceftazidime | 26 | 22 | 84.62 | 20 | 12 | 60 |
| Cefepime | 26 | 22 | 84.62 | 20 | 12 | 60 |
| Imipenem | 26 | 0 | 0 | 20 | 0 | 0 |
| Ciprofloxacin | 26 | 9 | 34.62 | 20 | 14 | 70 |
| Amikacin | 26 | 1 | 3.85 | 20 | 0 | 0 |
| Gentamicin | 26 | 10 | 38.46 | 20 | 13 | 65 |

**Table C. Antibiotic resistance proportion of *Klebsiella pneumoniae***

| **Antibiotics** | **2010-2013** | | |
| --- | --- | --- | --- |
|  | **total** | **resistance** | **Proportion (%)** |
| Ampicillin | 15 | 15 | 100 |
| Ampicillin/sulbactam | 14 | 8 | 57.14 |
| Piperacillin | 8 | 6 | 75 |
| Piperacillin/Tazobactam | 15 | 1 | 6.67 |
| Aztreonam | 15 | 8 | 53.33 |
| Ceftriaxone | 10 | 4 | 40 |
| Ceftazidime | 15 | 8 | 53.33 |
| Cefepime | 14 | 7 | 50 |
| Imipenem | 15 | 0 | 0 |
| Ciprofloxacin | 15 | 3 | 20 |
| Amikacin | 15 | 2 | 13.33 |
| Gentamicin | 15 | 8 | 53.33 |

**Table D. Antibiotic resistance proportion of *Enterobacter cloacae***

| **Antibiotics** | **2010-2013** | | |
| --- | --- | --- | --- |
|  | **total** | **resistance** | **Proportion (%)** |
| Ampicillin | 15 | 15 | 100 |
| Ampicillin/sulbactam | 14 | 13 | 92.86 |
| Piperacillin | 8 | 6 | 75 |
| Piperacillin/Tazobactam | 15 | 3 | 20 |
| Aztreonam | 15 | 5 | 33.33 |
| Ceftriaxone | 12 | 6 | 50 |
| Ceftazidime | 15 | 6 | 40 |
| Cefepime | 15 | 2 | 13.33 |
| Imipenem | 15 | 0 | 0 |
| Ciprofloxacin | 15 | 4 | 26.67 |
| Amikacin | 15 | 0 | 0 |
| Gentamicin | 15 | 4 | 26.67 |

**Table E. Antibiotic resistance proportion of *Pseudomonas aeruginosa***

| **Antibiotics** | **2009-2013** | | |
| --- | --- | --- | --- |
|  | **total** | **resistance** | **Proportion (%)** |
| Ampicillin | 7 | 7 | 100 |
| Ampicillin/sulbactam | 7 | 7 | 100 |
| Piperacillin | 17 | 4 | 23.53 |
| Piperacillin/Tazobactam | 23 | 3 | 13.04 |
| Aztreonam | 23 | 5 | 21.74 |
| Ceftriaxone | 22 | 10 | 45.45 |
| Cefoperazone | 1 | 1 | 100 |
| Ceftazidime | 23 | 5 | 21.74 |
| Cefepime | 23 | 5 | 21.74 |
| Imipenem | 23 | 4 | 17.39 |
| Ciprofloxacin | 23 | 8 | 34.78 |
| Amikacin | 23 | 4 | 17.39 |
| Gentamicin | 23 | 4 | 17.39 |

**Table F. Overall antibiotics usage frequency**

| Drug | #hosp | Proportion |
| --- | --- | --- |
| Levofloxacin | 112 | 20.25% |
| Penicillin | 99 | 17.90% |
| Cefoperazone and Sulbactam | 83 | 15.01% |
| Metronidazole | 83 | 15.01% |
| Gentamicin | 77 | 13.92% |
| Ornidazole | 76 | 13.74% |
| Cefuroxime | 67 | 12.12% |
| Cefoperazone and Tazobactam | 63 | 11.39% |
| Cefixime | 59 | 10.67% |
| Cefamandole | 55 | 9.95% |
| Nitrofurazone | 55 | 9.95% |
| Ceftriaxone and Tazobactam | 46 | 8.32% |
| Ciprofloxacin | 45 | 8.14% |
| Mupirocin | 43 | 7.78% |
| Amikacin | 41 | 7.41% |
| Cefathiamidine | 39 | 7.05% |
| Cefazolin | 39 | 7.05% |
| Ceftizoxime | 38 | 6.87% |
| Ceftriaxone | 36 | 6.51% |
| Piperacillin and Sulbactam | 36 | 6.51% |
| **Imipenem Cilastatin** | 35 | 6.33% |
| Cefodizime | 33 | 5.97% |
| Clindamycin | 33 | 5.97% |
| **Vancomycin** | 26 | 4.70% |
| Amoxicillin and Clavulanic Acid | 23 | 4.16% |
| **Norvancomycin** | 21 | 3.80% |
| Cefradine | 19 | 3.44% |
| Cefotiam | 18 | 3.25% |
| Amoxicillin | 17 | 3.07% |
| Moxifloxacin | 17 | 3.07% |
| Piperacillin and Tazobactam | 15 | 2.71% |
| Roxithromycin | 15 | 2.71% |
| Piperacillin Tazobactam | 14 | 2.53% |
| Chlortetracycline | 13 | 2.35% |
| Azithromycin | 12 | 2.17% |
| Ceftazidime | 12 | 2.17% |
| Latamoxef | 11 | 1.99% |
| **Meropenem** | **11** | **1.99%** |

**Table G. cSSTI infection types and microbiology. The number represents detected cases.**

| **Abscess** | |
| --- | --- |
| gram-positive | 12 |
| gram-negative | 16 |
| **Burn** | |
| gram-positive | 2 |
| **Cellulitis/fasciitis/erysipelas** | |
| gram-positive | 12 |
| gram-negative | 9 |
| **Diabetic leg ulcer** |  |
| gram-positive | 1 |
| **Post-surgical wound** | |
| gram-positive | 21 |
| gram-negative | 30 |
| **Post-traumatic wound** | |
| gram-negative | 4 |
| **Ulcer** |  |
| gram-positive | 3 |
| gram-negative | 3 |
| **Unknown** | |
| gram-positive | 25 |
| gram-negative | 16 |
